# RelA/MicroRNA-30a/NLRP3 signal axis is involved in rheumatoid arthritis via regulating NLRP3 inflammasome in macrophages

**DOI:** 10.1038/s41419-021-04349-5

**Published:** 2021-11-08

**Authors:** Qiudong Yang, Wenhua Zhao, Yuyi Chen, Yue Chen, Jiali Shi, Ran Qin, Hua Wang, Ruixia Wang, Hua Yuan, Wen Sun

**Affiliations:** 1 Department of Basic Science of Stomatology, The Affiliated Stomatological Hospital of Nanjing Medical University, Nanjing, China; 2 Department of Oral and Maxillofacial Surgery, The Affiliated Stomatological Hospital of Nanjing Medical University, Nanjing, China; 3 Jiangsu Province Key Laboratory of Oral Diseases, Nanjing, China; 4 Jiangsu Province Engineering Research Center of Stomatological Translational Medicine, Nanjing, China

**Keywords:** Mechanisms of disease, Inflammasome, Rheumatoid arthritis

## Abstract

NLRP3 inflammasome plays an important role in the pathogenesis of rheumatoid arthritis (RA). However, the post-transcriptional regulation of NLRP3 expression by miRNA in synovial macrophages is still not well understood. The aim of the study is to elucidate the mechanisms of RA with the focus on miRNAs mediated post-transcriptional regulation of the NLRP3 inflammasome. Here, we used NLRP3-deficient mice (NLRP3^KO^) to cross with TNFα-transgenic mice (TNF^TG^) to generate NLRP3^KO^/TNF^TG^ mice, and compared their joint phenotypes with those of their TNF^TG^ and wild-type (WT) littermates at 5 months of age. In comparison to WT mice, articular bone volume and cartilage area are decreased, whereas inflammed area, eroded surface, ALP+ osteoblast number, TRAP+ osteoclast number, and the areas of RelA+F4/80+, Caspase-1+F4/80+, IL-1β+F4/80+ synoviocytes are increased in the TNF^TG^ mice. Knockout of NLRP3 ameliorates joint inflammation and bone damage in TNF^TG^ mice. Further, in TNFα-primed BMDMs, RelA positively regulates NLRP3 expression, but negatively regulates miR-30a. Additionally, miR-30a negatively mediates NLRP3 expression by directly binding to its 3ʹ UTR, suggesting a miR-30a-mediated feedforward loop acting on NLRP3. Finally, intra-articular injection of AAV-miR-30a inhibits NLRP3 inflammasome activation, reduces joint inflammation, and attenuates bone damage in TNF^TG^ mice. Thus, RelA/miR-30a/NLRP3 signal axis is involved in RA through regulating NLRP3 Inflammasome in macrophages.

## Introduction

Rheumatoid arthritis (RA) is a chronic, destructive autoimmune inflammatory disorder, affecting 0.5–1% of the population worldwide [1]. It is one of the most common forms of arthritis which causes synovitis, bone erosion and joint deformity [2]. Many studies have shown a large number of pro-inflammatory cytokines are active in the joints of patients with RA [3, 4]. Among these cytokines, TNFα has gained much attention because of its position at the apex of the pro-inflammatory cytokine cascade, and its dominance in the pathogenesis of RA [5]. Therefore, a genetically modified TNFα-transgenic (TNF^TG^) mouse model, which over-expresses human TNFα and develops an erosive polyarthritis with many characteristics observed in RA patients, has been widely used in RA-related researches [6, 7]. However, the mechanisms underlying the progression of RA remain largely unknown.

In recent years, more attention has been paid to nucleotide-binding oligomerization domain (Nod)-like receptor family pyrin domain-containing 3 (NLRP3) inflammasome. NLRP3 inflammasome has been reported to play an important role in a variety of diseases including systemic lupus erythematosus, gout, atherosclerosis, type 2 diabetes, and RA [8–10]. The clinical evidence indicated that the activity of NLRP3 inflammasome of peripheral blood cells from patients with RA was more enhanced compared to that of the healthy control individuals [11]. A later study demonstrated synovial NLRP3 expression level was correlated with the clinical arthritis severity and extent of radiological destruction, suggesting that NLRP3 is involved in the pathogenesis of RA [12]. In addition, activation of NLRP3 inflammasome occurred mainly in the infiltrating monocytes/macrophages in synovia [13]. Therefore, we speculate that NLRP3 in monocytes/macrophages may exert major effects on RA-mediated synovitis and bone damage.

NLRP3 expression was shown to be post-transcriptionally regulated and multiple microRNAs (miRNA) have been implicated in post-transcriptional regulation of the NLRP3 inflammasome [14, 15]. MiRNAs are small non-coding RNAs with a length of 18–23 nucleotides that function as post-transcriptional regulators of gene expression [16]. MiRNAs interact directly with specific target mRNAs and cause the translational repression or degradation of the target genes [17]. MiRNA-based post-transcriptional control of NLRP3 has become a focus of much research, especially as a potential therapeutic approach [14]. Recently, a couple of miRNAs that mediated NLRP3 expression were found altered in RA, including miR-20a in T cells [18] and miR-223 in fibroblast-like synoviocytes [19]. However, NLRP3 is mainly expressed in macrophages [20] and the post-transcriptional regulation of NLRP3 expression by miRNA in synovial macrophages is still not well understood in RA.

The current study seeks to elucidate the mechanisms of synovitis and bone damage in RA with the focus on miRNAs mediated post-transcriptional regulation of the NLRP3 inflammasome. We used TNF^TG^ mouse as RA murine model, and found that knockout of NLRP3 ameliorated joint inflammation and bone damage in TNF^TG^ mice. We further provided a direct link among RelA, miR-30a and NLRP3 inflammasome, suggesting a miR-30a-mediated feedforward loop acting on NLRP3. At last, local miR-30a overexpression decreased NLRP3 activation, and further protected against joint inflammation and bone damage in RA mice. Thus, our findings reveal a previously unappreciated role for miR-30a in RA-associated synovitis and bone damage via direct regulation of the NLRP3 inflammasome in synovial macrophages.

## Materials and methods

### Mice

NLRP3-deficient (NLRP3^KO^) mice generated in a C57BL/6J background were kindly provided by Professor Shuo Yang [21]. TNF^TG^ mice (line 3647) were originally obtained from Dr. G. Kollias (Institute of Immunology, Alexander Fleming Biomedical Sciences Research Center, Vari, Greece) and have been crossed with C57BL/6J mice for more than 10 generations [22]. NLRP3^+/−^ female mice and TNF^TG^ male mice were crossed to generate NLRP3^+/−^; TNF^TG^ double-mutant mice, and NLRP3^+/−^; TNF^TG^ males and NLRP3^+/−^ females were crossed to generate NLRP3^KO^/TNF^TG^ mice. Two-month-old or five-month-old male wild-type (WT), NLRP3^KO^, TNF^TG^, and NLRP3^KO^/TNF^TG^ mice (*N* = 5 or 10) were used in the current study, as specified in figure legends. All animals were randomized for their genotype information and were included in the study. All mice were bred and maintained in the SPF Laboratory Animal Center of Nanjing Medical University. The use of animals in this study was approved by the Institutional Animal Care and Use Committee of Nanjing Medical University (Approval ID1906018). Additional supporting information may be found in Supplementary Methods on line.

## Results

### NLRP3 deficiency ameliorates joint inflammation and bone damage in TNF^TG^ RA mice

**Fig. 1:**
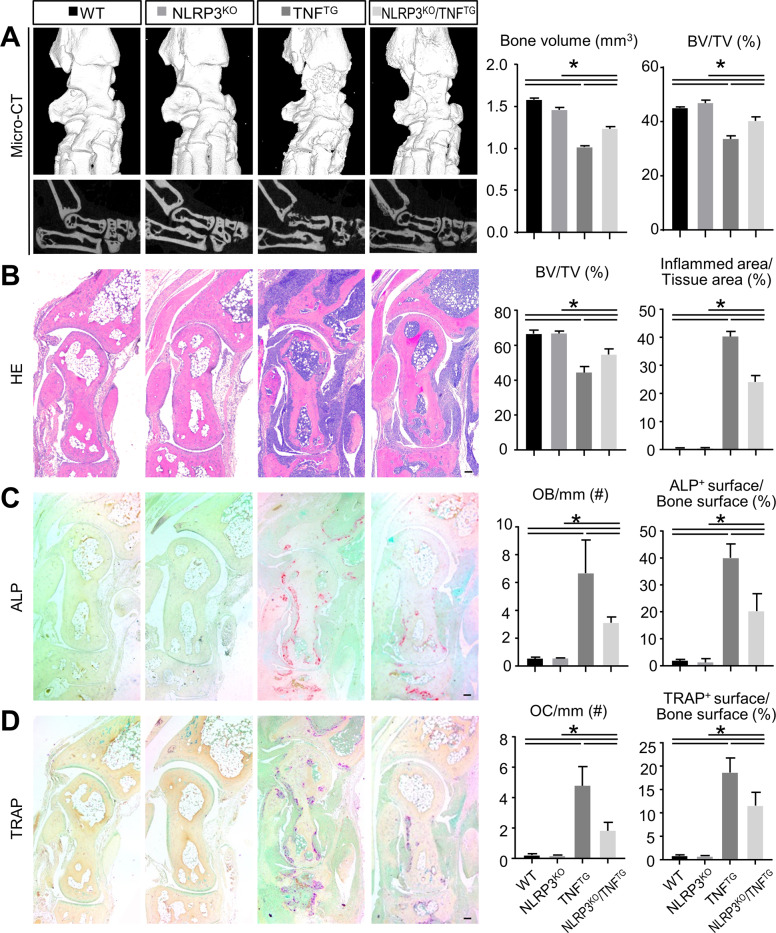
NLRP3 deficiency ameliorates joint inflammation and bone damage in TNF^TG^ RA mice. Five-month-old WT, NLRP3^KO^, TNF^TG^, NLRP3^KO^/TNF^TG^ mice were used. **A** Representative reconstructed sections (upper panels) and 3D scanned sections (lower panels) of ankle joints and morphometric data of talus bone volume (mm^3^) and articular bone volume relative to tissue volume (%). **B** Representative images of H&E-stained paraffin sections show ankle joints. Articular bone volume relative to tissue volume (%) and inflammed area relative to tissue area (%) were determined. **C** Representative images of ALP-stained paraffin sections show ankle joints. Number of ALP-positive osteoblasts relative to bone surface (#/mm) and the surface of osteoblasts relative to the bone surface (%) were determined. **D** Representative images of TRAP-stained paraffin sections show ankle joints. Number of TRAP-positive osteoclasts relative to bone surface (#/mm) and the surface of osteoclasts relative to the bone surface (%) were determined. Scale bar = 100 μm. All error bars represent SD. One-way ANOVA followed by Dunnett’s post-hoc multiple comparisons was performed. *N* = 5, *P < 0.05 in the indicated groups.

To explore the role of NLRP3 in RA pathogenesis, we deleted NLRP3 gene in TNF^TG^ RA mice to generate NLRP3^KO^/TNF^TG^ mice, their joint phenotypes were compared with those of NLRP3^KO^, TNF^TG^, and WT mice. In comparison to WT mice, micro-CT and histomorphometric analyses of ankles showed a similar bone volume in NLRP3^KO^ mice, but a decreased bone volume in TNF^TG^ mice and NLRP3^KO^/TNF^TG^ mice (Fig. 1A). The inflammed area was significantly increased in TNF^TG^ mice and NLRP3^KO^/TNF^TG^ mice compared with that of the WT mice (Fig. 1B). However, both the bone volume and the inflammed area were clearly rescued in NLRP3^KO^/TNF^TG^ mice compared with those of the TNF^TG^ mice. These results were also confirmed using knee joints of WT, NLRP3^KO^, TNF^TG^ and NLRP3^KO^/TNF^TG^ mice (Fig. S1).

We next examined osteoblastic bone formation in paraffin-embedded sections stained histochemically for ALP. The number of osteoblasts and the ALP-positive surface were determined with image analysis. The number of osteoblasts and the ALP-positive surface were significantly increased in TNF^TG^ mice and NLRP3^KO^/TNF^TG^ mice compared with those of the WT mice (Fig. 1C). Both the parameters were partly rescued in NLRP3^KO^/TNF^TG^ mice compared with TNF^TG^ mice (Fig. 1C). Next, osteoclastic bone resorption was determined in paraffin-embedded sections stained with TRAP. Histomorphometric analysis showed that the number of osteoclasts and the TRAP-positive surface were significantly increased in TNF^TG^ mice and NLRP3^KO^/TNF^TG^ mice compared with those of the WT mice (Fig. 1D). Both the parameters were partly rescued in NLRP3^KO^/TNF^TG^ mice compared with TNF^TG^ mice (Fig. 1D). Therefore, these results indicated that knockout of the NLRP3 gene ameliorates joint inflammation and bone damage in TNF^TG^ RA mice.

### NLRP3 deficiency attenuates joint erosion and cartilage degradation in TNF^TG^ RA mice

**Fig. 2:**
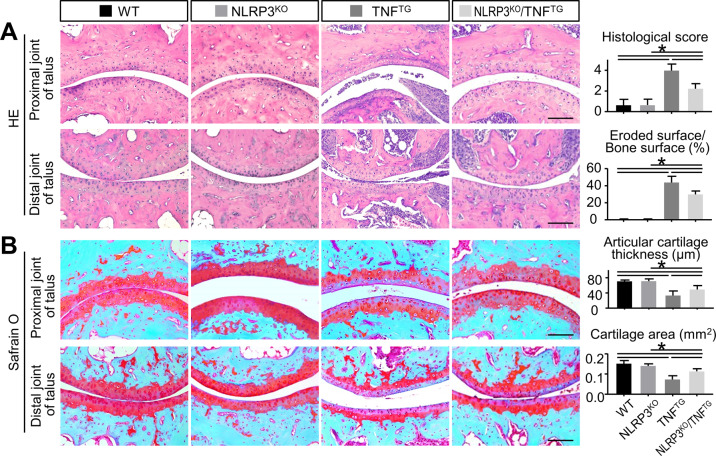
NLRP3 deficiency attenuates joint erosion and cartilage degradation in TNF^TG^ RA mice. Five-month-old WT, NLRP3^KO^, TNF^TG^, NLRP3^KO^/TNF^TG^ mice were used. **A** Representative images of H&E-stained paraffin sections show the articular surface in ankle joints. Both proximal and distal joints of talus are shown. Histological arthritis score and the eroded surface relative to the bone surface (%) were determined. **B** Representative images of Safranin O-stained paraffin sections show the articular cartilage degradation in ankle joints. Both proximal and distal joints of talus are shown. Articular cartilage thickness (μm) and cartilage area (mm^2^) were determined. Scale bar = 100 μm. All error bars represent SD. One-way ANOVA followed by Dunnett’s post-hoc multiple comparisons was performed. *N* = 5, *P < 0.05 in the indicated groups.

To further determine the effects of NLRP3 deficiency on joint erosion in TNF^TG^ RA mice, paraffin sections were stained with H&E staining and subjected to histomorphometric analyses. The histological arthritis score and the eroded surface were increased significantly in TNF^TG^ mice and NLRP3^KO^/TNF^TG^ mice compared with those of the WT mice (Fig. 2A). However, both the parameters were partly rescued in NLRP3^KO^/TNF^TG^ mice compared with TNF^TG^ mice (Fig. 2A).

We next examined cartilage degradation in paraffin-embedded sections stained histochemically with Safrain O. The thickness and the area of articular cartilage were decreased clearly in TNF^TG^ mice and NLRP3^KO^/TNF^TG^ mice compared with those of the WT mice (Fig. 2B and Fig. S1). However, both the parameters were partly rescued in NLRP3^KO^/TNF^TG^ mice compared with TNF^TG^ mice (Fig. 2B and Fig. S1). These findings demonstrated that NLRP3 deletion partly prevents joint erosion and cartilage degradation in TNF^TG^ RA mice.

### NLRP3 deficiency inhibits the activation of NF-κB/NLRP3 signaling molecules in synovial macrophages

**Fig. 3:**
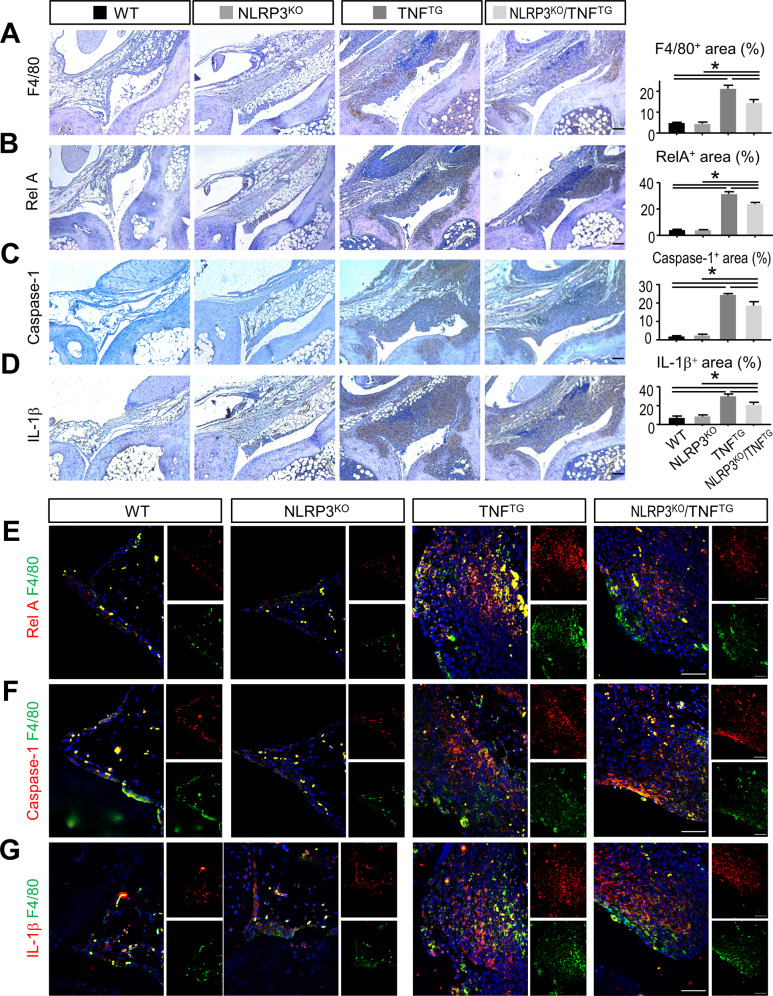
NLRP3 deficiency inhibits the activation of NF-κB/NLRP3 signaling molecules in synovial macrophages. Five-month-old WT, NLRP3^KO^, TNF^TG^, NLRP3^KO^/TNF^TG^ mice were used. **A**–**D** Adjacent paraffin sections were stained immunohistochemically for F4/80 (**A**), RelA (**B**), Caspase-1 (**C**) and IL-1β (**D**). Representative images of ankle synovium are shown. The percentage of F4/80, RelA, Caspase-1 and IL-1β positive area were calculated, respectively. **E**–**G** Adjacent paraffin sections were further subjected to immunofluorescence staining with anti-F4/80 and anti-RelA (**E**) or Caspase-1 (**F**) or IL-1β (**G**) Abs. Representative images of ankle synovium are shown. Scale bar = 100 μm. All error bars represent SD. One-way ANOVA followed by Dunnett’s post-hoc multiple comparisons was performed. *N* = 5, *P < 0.05 in the indicated groups.

TNFα overexpression triggers the activation of transcription factor RelA via the canonical NF-κB pathway, which induces NLRP3 and IL-1β expression at the transcriptional level and then enhances the activation of NLRP3 inflammasome [23]. NLRP3 inflammasome activation further mediates Caspase-1 activation and IL-1β maturation [23, 24]. To explore the mechanisms responsible for the improved joint inflammation and bone damage in NLRP3^KO^/TNF^TG^ mice compared with TNF^TG^ mice, we examined the expression of NF-κB/NLRP3 signaling molecules using paraffin-embedded sections of ankle joint by immunohistochemical staining. The data revealed that the areas of F4/80+ macrophages and RelA+ synoviocytes were significantly increased in TNF^TG^ mice and NLRP3^KO^/TNF^TG^ mice compared with those of the WT mice (Fig. 3A, B). Similarly, the areas of Caspase-1+ and IL-1β+ synoviocytes both evidently greater in ankle synovium from TNF^TG^ mice and NLRP3^KO^/TNF^TG^ mice than in those from WT mice (Fig. 3C, D). More importantly, all the parameters were partly rescued in NLRP3^KO^/TNF^TG^ mice compared with TNF^TG^ mice (Fig. 3A–D). In addition, most of the RelA + , Caspase-1+ or IL-1β+ synoviocytes might co-localize with F4/80+ macrophages, suggesting the majority of cells carrying activated NF-κB/NLRP3 signaling in TNF^TG^ RA synovium may be macrophages.

To confirm these data, double IF staining was performed using paraffin sections of ankle joints. We observed numerous RelA+F4/80+, Caspase-1+F4/80+, and IL-1β+F4/80+ macrophages in synovial sections of TNF^TG^ mice (Fig. 3E–G). However, RelA+F4/80+, Caspase-1+F4/80+, and IL-1β+F4/80+ macrophages were decreased in NLRP3^KO^/TNF^TG^ mice compared with TNF^TG^ mice (Fig. 3E-G). Therefore, these data indicate that NLRP3 deficiency inhibits the activation of NF-κB/NLRP3 signaling molecules in synovial macrophages.

### RelA mediates TNFα−induced miR-30a expression and NLRP3 inflammasome activation

**Fig. 4:**
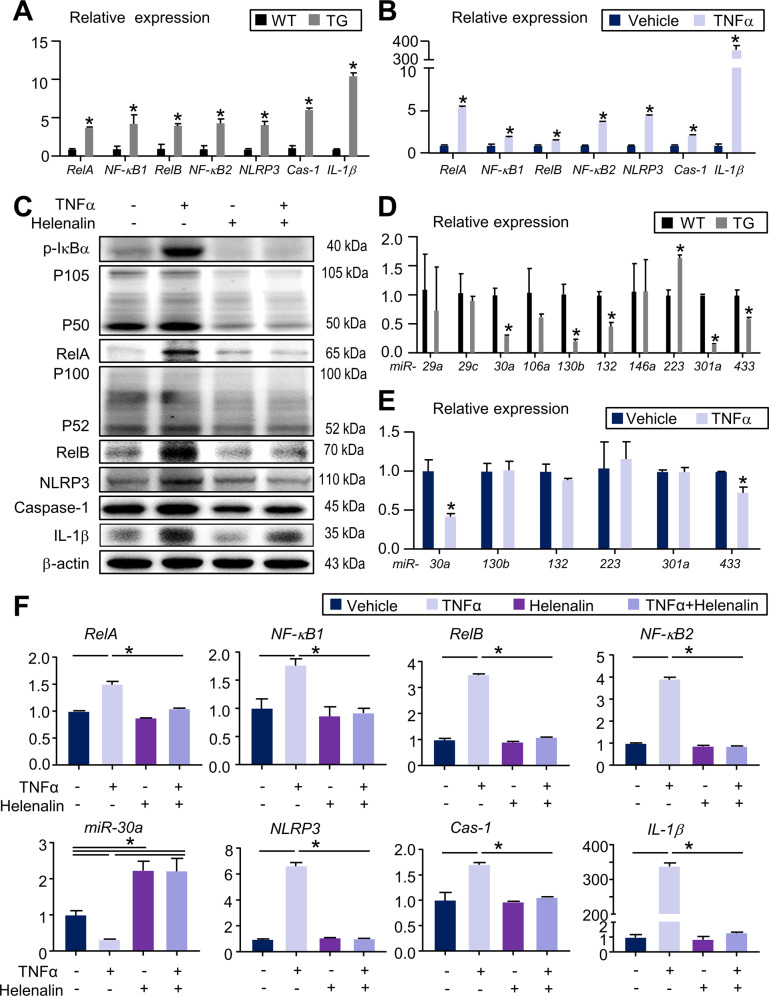
RelA mediates TNFα-induced miR-30a expression and NLRP3 inflammasome activation. **A** The relative gene expression levels of *RelA*, *NF-kB1*, *RelB*, *NF-kB2*, *NLRP3*, *Cas-1*, and *IL-1β* in synovial tissues of 5-month-old WT and TNF^TG^ mice were determined by qPCR. **B** Bone marrow cells from 2-month-old WT mice were cultured with M-CSF for 3 days to generate BMDMs. BMDMs were treated with or without 20 ng/mL TNFα and then subjected to qPCR. The relative gene expression levels of *RelA*, *NF-kB1*, *RelB*, *NF-kB2*, *NLRP3*, *Cas-1*, and *IL-1β* were determined. **C** BMDMs were treated with or without 20 ng/mL TNFα ± 10μM helenalin. The relative expression levels of p-IκBα, p50/105, RelA, p52/100, RelB, NLRP3, Caspase-1 and IL-1β were determined by Western blot analysis. Supplementary Information shows uncropped gel images. **D** The relative gene expression levels of *miR-29a, miR-29c, miR-30a, miR-106a, miR-130b, miR-132, miR-146a, miR-223, miR-301a*, and *miR-433* in synovial tissues of 5-month-old WT and TNF^TG^ mice were determined by qPCR. **E** BMDMs were treated with or without 20 ng/mL TNFα and then subjected to qPCR. The relative gene expression levels of *miR-30a, miR-130b, miR-132, miR-223, miR-301a*, and *miR-433* were determined. **F** BMDMs were treated with or without 20 ng/mL TNFα ± 10μM Helenalin. The relative expression levels of *RelA*, *NF-kB1*, *RelB*, *NF-kB2*, *miR-30a, NLRP3*, *Cas-1*, and *IL-1β* were determined by qPCR. The primer sequences used for the qPCR are shown in Supplementary Table 1. All the results are representative of at least three independent experiments. Values are mean ± SD. Two-tailed unpaired Student’s *t* test or One-way ANOVA followed by Dunnett’s post-hoc multiple comparisons was performed. *P < 0.05 vs. WT/Veh or in the indicated groups.

To further examine the NF-κB/NLRP3 signaling molecules in response to TNFα overexpression, RNA was isolated from synovial tissues of ankle joints and TNFα-primed BMDMs to test the gene expression of *RelA, NF-κB1*, *RelB, NF-κB2, NLRP3, Caspase-1* and *IL-1β*. Results showed that mRNA levels of *RelA, NF-κB1*, *RelB, NF-κB2, NLRP3, Caspase-1* and *IL-1β* were significantly upregulated in the TNF^TG^ mice as compared with their WT littermates (Fig. 4A). Similarly, mRNA levels of these genes were also increased in TNFα-induced BMDMs (Fig. 4B). Given the markedly increased level of TNFα-induced RelA (Fig. 3B, E and Fig. 4A, B), which may mediate NLRP3 inflammasome activation [23], we next examined for changes of NF-κB/NLRP3 signaling molecules in BMDMs with or without helenalin, a selective inhibitor of RelA [25]. By Western blot analysis, the expression levels of NF-κB proteins, NLRP3, Caspase-1, and IL-1β were increased in TNFα-treated BMDMs (Fig. 4C). In addition, RelA inhibition by helenalin completely inhibited the NF-κB and NLRP3 activation in these cells (Fig. 4C).

Since *NLRP3* gene expression can be governed by miRNAs via post-transcriptional regulation, we next investigated the expression of miRNAs in ankle joints of TNF^TG^ mice and their WT littermates by qPCR with the focus on highly conserved monocyte/macrophage-specific miRNAs [26]. We used several established miRNA-target prediction programs (*miRwalk, miRanda, RNA22, TargetScanv6.2, mirDIP* and *miRDB*) to identify miRNAs that may target *NLRP3* mRNA, including *miR-29a, miR-29c, miR-30a, miR-106a, miR-130b, miR-132, miR-146a, miR-223, miR-301a*, and *miR-433*. Interestingly, qPCR analysis showed that the levels of *miR-30a, miR-130b, miR-132, miR-301a* and *miR-433* in ankle joints of TNF^TG^ mice were significantly lower than those in their WT littermates (Fig. 4D). In contrast, the level of *miR-223* was increased in TNF^TG^ mice compared with that in WT littermates (Fig. 4D). To further evaluate the functional miRNA that targets *NLRP3* mRNA, we used qPCR to examine the levels of altered miRNAs mentioned above in TNFα-primed BMDMs. Both *miR-30a* and *miR-433* were significantly decreased in TNFα-primed BMDMs (Fig. 4E). Importantly, the most differentially expressed miRNA between TNFα-primed BMDMs and vehicle-treated BMDMs was *miR-30a*, suggesting *miR-30a* is the most promising candidate for further investigation (Fig. 4E).

To further test if RelA mediates TNFα-induced *miR-30a* expression and NLRP3 inflammasome activation, RNA was isolated from TNFα-primed BMDMs treated with or without helenalin to test the gene expression of *miR-30a* and NF-κB/NLRP3 signaling molecules. As expected, *miR-30a* was significantly decreased in TNFα-primed BMDMs, and the changes were completely blocked by the RelA-specific inhibitor, helenalin (Fig. 4F). By contrast, the expression levels of *RelA, NF-κB1*, *RelB, NF-κB2, NLRP3, Caspase-1* and *IL-1β* were all significantly upregulated in TNFα-primed BMDMs, and the changes were completely blocked by helenalin (Fig. 4F). Taken together, these data suggest that RelA mediates TNFα-induced *miR-30a* expression and NLRP3 inflammasome activation.

### MiR-30a suppresses NLRP3 inflammasome activation by directly binding to the 3ʹ-UTR of NLRP3

**Fig. 5:**
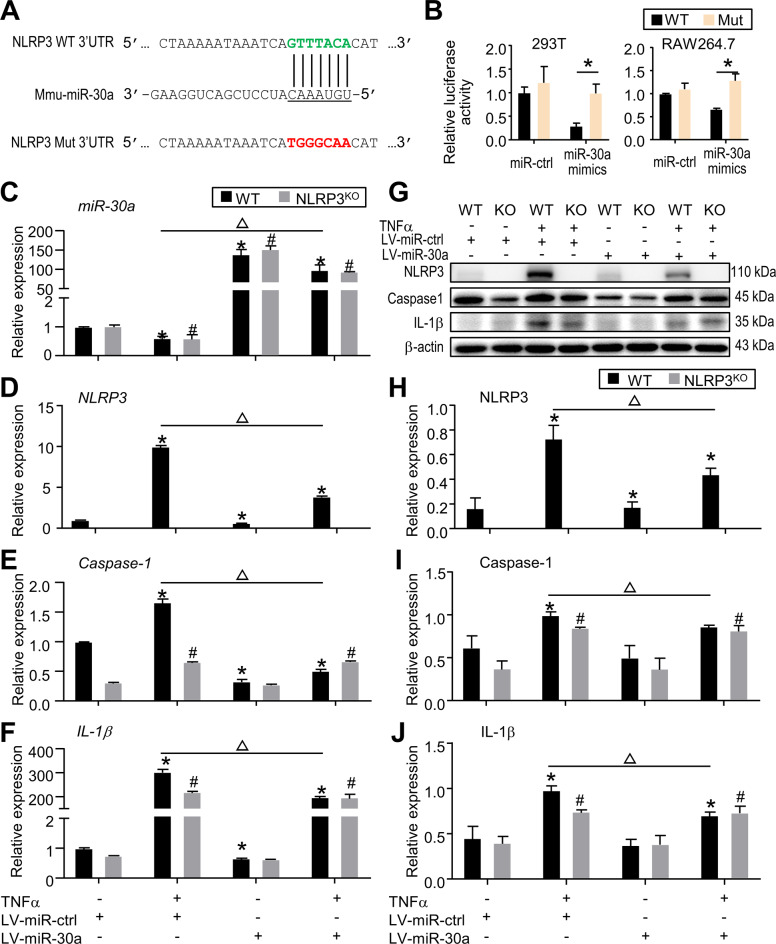
MiR-30a suppresses NLRP3 inflammasome activation by directly binding to the 3ʹ-UTR of NLRP3. **A** Predicted miR-30a binding site in the 3ʹ untranslated region (UTR) of NLRP3. The seed sequences and mutant sequences of NLRP3 were highlighted in green and red, respectively. **B** The wild-type (WT) or mutant (Mut) reporter constructs was co-transfected with control (miR-ctrl) or miR-30a mimics into HEK293T or RAW264.7 cells, and the dual luciferase activity was determined at 24 h after transfection. All the results are representative of at least three independent experiments. Values are mean ± SD. Two-tailed unpaired Student’s *t* test was performed. *P < 0.05 in the indicated groups. **C**–**J** BMDMs from NLRP3^KO^ mice and their WT littermates were generated as in Fig. 4. BMDMs were transfected with control or miR-30a lentivirus (LV-miR-Ctrl or LV-miR-30a), and then stimulated with or without 20 ng/ml TNFα during culture. **C**–**F** The relative gene expression levels of *miR-30a* (**C**), *NLRP3* (**D**), *Caspase-1* (**E**) and *IL-1β* (**F**) were determined by qPCR. **G**–**J** Protein lysates were subjected to Western blot analyses. Supplementary Information shows uncropped gel images. The relative expression levels of NLRP3 (**H**), Caspase-1 (**I**) and IL-1β (**J**) in cell lysates were assessed. All the results are representative of at least three independent experiments. Values are mean ± SD. One-way ANOVA followed by Dunnett’s post-hoc multiple comparisons was performed. *P < 0.05 vs. WT without treatment, ^#^*P* < 0.05 vs. NLRP3^KO^ without treatment, or ^△^*P* < 0.05 in the indicated groups.

To further verify the close connection and ascertain the underlying mechanism, we predicted the miR-30a binding site in the 3ʹ untranslated region (UTR) of NLRP3 via prediction programs mentioned above (Fig. 5A). After co-transfecting WT or mutant NLRP3 3ʹ UTR with a miR-30a mimic or miR-ctrl in the HEK293T cells, a dual-luciferase reporter assay was carried out. The results showed a much lower luciferase reporter activity in WT NLRP3 3ʹ UTR overexpressed with miR-30a than in mutant NLRP3 3ʹ UTR overexpressed with miR-30a (Fig. 5B). These data were also confirmed by dual-luciferase reporter assay using RAW264.7 cell, an established macrophage-like cell line (Fig. 5B). Since *miR-433* was also significantly decreased in TNFα-primed BMDMs and in ankle joints of TNF^TG^ mice (Fig. 4D, E), we examined the correlation between miR-433 and NLRP3 using dual-luciferase reporter assay. However, unlike miR-30a, we didn’t observe the changes of luciferase reporter activity in WT NLRP3 3ʹ UTR overexpressed with miR-433 and in mutant NLRP3 3ʹ UTR overexpressed with miR-433 (Fig. S2). Altogether, these data demonstrate that miR-30a specifically binds to the NLRP3 3ʹ-UTR to dampen the NLRP3 expression.

To determine if overexpressed miR-30a could block NLRP3 inflammasome activation in TNFα-primed BMDMs, thereby attenuating joint tissue damage in RA, we infected BMDMs from WT or NLRP3^KO^ mice with miR-30a lentivirus. In TNFα-primed BMDMs from WT mice, miR-30a overexpression significantly downregulated *NLRP3, Caspase-1* and *IL-1β* mRNA levels, which were not observed in TNFα-primed BMDMs from NLRP3^KO^ mice (Fig. 5C–F). These data were further confirmed by Western blot analysis, the expression levels of NLRP3, Caspase-1, and IL-1β were increased in TNFα-treated BMDMs from WT mice, which were blocked by miR-30a overexpression (Fig. 5G–J). In addition, the increased levels of NLRP3, Caspase-1, and IL-1β were not changed in TNFα-treated BMDMs from NLRP3^KO^ mice treated with or without miR-30a lentivirus (Fig. 5G–J). Therefore, these data indicate that miR-30a overexpression could block NLRP3 inflammasome activation, which was completely blocked by NLRP3 deficiency.

### AAV-miR-30a inhibits NLRP3 inflammasome activation, reduces joint inflammation, and attenuates bone damage in TNF^TG^ RA mice

**Fig. 6:**
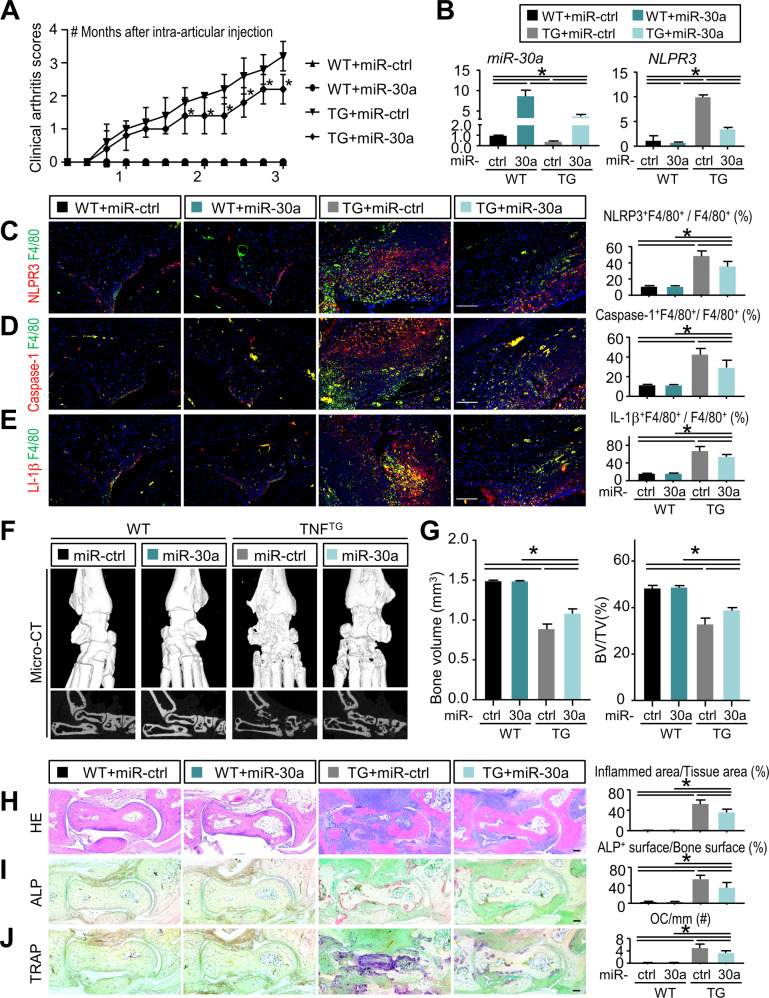
AAV-miR-30a inhibits NLRP3 inflammasome activation, reduces joint inflammation, and attenuates bone damage in TNF^TG^ RA mice. Two-month-old TNF^TG^ mice and their WT littermates received intra-articular injection of adeno-associated virus encoding miR-ctrl (left ankle) or miR-30a (right ankle) into joint cavities of the left or right ankle, respectively. Three months after viral injection, mice were sacrificed and analyzed. **A** Clinical arthritis scores were determined every week after viral injection for 3 months. Values are mean ± SD. One-way ANOVA followed by Dunnett’s post-hoc multiple comparisons was performed. *P < 0.05 vs. TG + miR-ctrl, at the same time point. **B** The relative gene expression levels of *miR-30a* and *NLRP3* in synovial tissues were determined by qPCR. **C** Adjacent paraffin sections were subjected to double immunofluorescence staining with anti-F4/80 and anti-NLRP3 (**C**) or Caspase-1 (**D**) or IL-1β (**E**) Abs. Representative images of ankle synovium are shown. The proportion of NLPR3+F4/80+, Caspase-1+F4/80+, and IL-1β+F4/80+ cells to F4/80+ cells (%) were determined. **F** Representative micro-CT images of reconstructed sections (upper panels) and 3D scanned sections (lower panels) of ankle joint are shown. **G** Morphometric data of talus bone volume (mm^3^) and articular bone volume relative to tissue volume (%) were calculated. **H** Representative images of H&E-stained paraffin sections show ankle joints. Inflammed area relative to tissue area (%) was determined. **I** Representative images of ALP-stained paraffin sections show ankle joints. The surface of ALP + osteoblasts relative to the bone surface (%) was determined. **J** Representative images of TRAP-stained paraffin sections show ankle joints. Number of TRAP-positive osteoclasts relative to bone surface (#/mm) was determined. Scale bar = 100 μm. All error bars represent SD. One-way ANOVA followed by Dunnett’s post-hoc multiple comparisons was performed. *N* = 10, *P < 0.05 in the indicated groups.

To determine if overexpressed miR-30a attenuates joint inflammation and bone damage in TNF^TG^ RA mice, 2-month-old TNF^TG^ mice and their WT littermates were intra-articular injected adeno-associated virus (AAV) encoding miR-30a or miR-ctrl into the joint cavities of ankle. All the mice were euthanized 3 months post-injection, when they typically had severe joint inflammation and bone damage (Fig. 1). Compared with the TNF^TG^ mice receiving AAV-miR-ctrl, ankle joints of TNF^TG^ mice receiving AAV-miR-30a had markedly declined clinical arthritis scores (Fig. 6A). Next, the overexpression of *miR-30a* was determined by qPCR using synovial tissues of ankle joint from TNF^TG^ mice receiving AAV-miR-30a compared with TNF^TG^ mice receiving AAV-miR-ctrl (Fig. 6B). In response to upregulated *miR-30a*, the expression level of *NLRP3* was significantly downregulated in the synovial tissues of ankle joint from TNF^TG^ mice receiving AAV-miR-30a compared with that from TNF^TG^ mice receiving AAV-miR-ctrl (Fig. 6B). Since AAV vectors used in this study also encode GFP, unstained frozen sections of ankle joint were directly observed under fluorescence microscopy, which further confirmed that AAV-mediated gene delivery to synovium was successful (Fig. S3). By IF, the proportion of NLPR3+, Caspase-1+, and IL-1β+ macrophages decreased significantly in ankle synovium of TNF^TG^ mice receiving AAV-miR-30a compared with TNF^TG^ mice receiving AAV-miR-ctrl (Fig. 6C–E).

To examine if suppressed NLRP3 inflammasome by AAV-miR-30a injection had a beneficial effect on RA-associated joint inflammation and bone damage, micro-CT and morphometric analyses were performed on the mouse ankle. Three-dimensional reconstruction images and volumetric measurements from micro-CT analysis revealed that the bone volume was improved in the ankle of TNF^TG^ mice receiving AAV-miR-30a compared with TNF^TG^ mice receiving AAV-miR-ctrl (Fig. 6F, G). Furthermore, morphometric measurements of H&E-stained tissue sections showed that the inflammed area was significantly decreased in TNF^TG^ mice receiving AAV-miR-30a compared with TNF^TG^ mice receiving AAV-miR-ctrl (Fig. 6H). In addition, both ALP-positive osteoblast surface and TRAP-positive osteoclast number were decreased in TNF^TG^ mice receiving AAV-miR-30a compared with TNF^TG^ mice receiving AAV-miR-ctrl (Fig. 6I, J). Taken together, these data indicate that AAV-miR-30a inhibits NLRP3 inflammasome activation, reduces joint inflammation, and attenuates bone damage in TNF^TG^ RA mice.

## Discussion

MiRNA-based post-transcriptional control of NLRP3 has become a focus of much research. A previous study demonstrated miR-223 negatively regulates NLRP3 inflammasome activation by directly binding to its 3ʹ UTR in macrophage [27]. However, this study didn’t test whether dysregulation of miR-223 expression plays a role in the pathogenesis of inflammatory diseases in vivo. Another study found that miR-7 targets NLRP3 expression, while miR-7 mimics suppress NLRP3 inflammasome activation and protects dopaminergic neurons against degeneration in a murine model of Parkinson’s disease [28]. However, many studies have shown that miR-7 is highly enriched in brain tissue and is closely related to physiological and pathological processes of brain [29–31], suggesting miR-7 may not be a good candidate for the research or the therapeutic target for RA. In this study, we computationally identified 10 highly conserved monocyte/macrophage-specific miRNAs that may target NLRP3, including miR-29a, miR-29c, miR-30a, miR-106a, miR-130b, miR-132, miR-146a, miR-223, miR-301a, and miR-433. Using a series of bioinformatic analyses and experimental tests, miR-30a was finally demonstrated as a negative regulator of NLRP3 by directly binding to its 3ʹ UTR in macrophage.

**Fig. 7:**
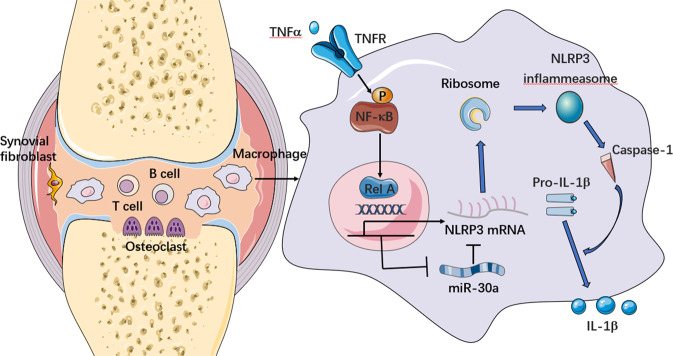
Schematic representation of the RelA/miR-30a/NLRP3 signal axis in synovial macrophages from RA joints. In synovial macrophages from RA joints, TNFα overexpression triggers the activation of transcription factor RelA via the canonical NF-κB pathway. Next, RelA positively regulates NLRP3 expression, but negatively regulates miR-30a. Additionally, miR-30a negatively mediates NLRP3 expression by directly binding to its 3ʹ UTR, suggesting a miR-30a-mediated feedforward loop acting on NLRP3. Finally, increased expression of NLRP3 enhances the activation of NLRP3 inflammasome.

It has been reported that RelA positively regulates the transcription of NLRP3 and promotes the formation of the NLRP3 inflammasome in macrophages [32], which was also confirmed in the current study. In addition, our data demonstrated that RelA modulates the expression of miR-30a in macrophages by recognizing specific sites in the regulatory elements of miR-30a gene. These results suggested the transcription rate of NLRP3 and miR-30a are co-regulated by RelA, that is NLRP3 is positively regulated by RelA, but miR-30a is negatively regulated by RelA. Additionally, MiR-30a further negatively regulates NLRP3 expression by directly binding to its 3ʹ UTR, suggesting a miR-30a-mediated feedforward loop acting on NLRP3 (Fig. 7). Thus, miR-30a regulates NLRP3 coherently with transcriptional control, thereby reinforcing transcriptional logic at the post-transcriptional level. As reported, under conditions where the target genes are transcriptionally promoted, such feedforward loops can serve as a surveillance mechanism to suppress “leaky” transcription of target genes [33]. Taken together, our results demonstrated RelA mediates the expression of NLRP3 directly by transcriptional regulation and indirectly by post-transcriptional regulation through miR-30a as a feedforward manner.

For clinical relevance of our mouse findings, we have tried to find the correlation between miR-30a and NLRP3 based on publicly available human RA dataset. However, we failed to find any human RA datasets reporting the expression profiles of miRNA and messenger RNA together. It has been reported that miR-30a expression is downregulated in inflammatory lesions of human RA using comparative miRNA screenings [34]. In addition, Real-time PCR was performed to show a significant decrease of miR-30a in RA synovial tissues when compared with osteoarthritis (OA) [35]. Since NLRP3 was also closely related to RA [36–38], the expression levels of *miR-30a* and *NLRP3* in synovial tissues of RA patients were assessed based on the data from public databases, respectively. As expected, the expression level of *miR-30a* was significantly downregulated in synovial tissues of RA patients compared with representative controls (Fig. S4). On the contrary, the expression level of *NLRP3* was significantly upregulated in synovial tissues of RA patients compared with representative controls (Fig. S4). Therefore, we may pay attention to the correlation between miR-30a and NLRP3 in macrophages from RA patients in the future.

With the in-depth research on the mechanism of NLRP3 inflammasome, a variety of small molecule compounds have been found to act as inhibitors of NLRP3 inflammasome, such as MCC950, CY-09, INF39. Among these, MCC950 is the most widely verified for the treatment of many inflammatory diseases, including RA [13], atherosclerosis [39], Alzheimer’s disease [40], and colitis [41]. However, the application of MCC950 is limited by liver toxicity based on phase II clinical trial for RA [42]. Therefore, developing new treatment strategies with the focus on NLRP3 inflammasome to prevent RA progression is crucial. In the current study, we have described the protective effects of miR-30a, in an animal model of RA. A very recent study also reported that miR-30a ameliorates oxidative stress in RA synovial fibroblasts via activation of Nrf2-ARE signaling pathway. Furthermore, our data indicated that AAV-miR-30a inhibits NLRP3 inflammasome activation, reduces joint inflammation, and attenuates bone damage in TNF^TG^ RA mice. Since miR-30a is highly conserved between human and mouse [26], we believe that miR-30a may serve as a potential therapeutic approach to prevent RA-associated joint inflammation and bone damage.

Notably, although locally administered miR-30a reduced joint inflammation and bone damage, the effects were incomplete, suggesting that either the concentration of miR-30a attained locally was insufficient or, more likely, other factors contribute to the joint inflammation and bone damage. Another concern is that although NLRP3 is mainly expressed in monocytes/macrophages [43], it is also expressed in mesenchymal stem cells [44], osteoblasts [45], osteoclasts [46], and synovial fibroblasts [47]. It would be beneficial to generate myeloid-specific NLRP3-overexpression or knockout mice by crossing NLRP3^TG^ mice or NLRP3-floxed mice with LysM-cre or CX3CR1-cre mice and then inducing RA by injecting mice with type II collagen in the absence and presence of AAV-miR-30a administration. Such studies could define even better the role of miR-30a interact with NLRP3 in macrophages and its effect on RA treatment.

In summary, using a murine model of RA and TNFα-primed macrophages, we provide a direct link among RelA, miR-30a and NLRP3 inflammasome-mediated joint inflammation and bone damage in the pathogenesis of RA. Local miR-30a overexpression decreased NLRP3 activation, and further protected against joint inflammation and bone damage in RA mice. Thus, our findings suggest RelA/miR-30a/NLRP3 signal axis is involved in RA through regulating NLRP3 inflammasome in macrophages.

## Supplementary information

Supplementary Methods

Figure S1

Figure S2

Figure S3

Figure S4

Supplementary Table

Supplementary Information- Uncropped full Western blot scans

## Data Availability

The authors declare that all data supporting the findings of this study are available within the article or are available from the corresponding author upon request.
