# Supplementary material for: RelA/MicroRNA-30a/NLRP3 signal axis is involved in rheumatoid arthritis via regulating NLRP3 inflammasome in macrophages
Source: Cell Death Dis. 2021 Nov 8;12(11):1060. doi: 10.1038/s41419-021-04349-5 (PMC8575917; doi:10.1038/s41419-021-04349-5)
Supplement: Supplementary file 1 — Supplementary Methods [file 41419_2021_4349_MOESM1_ESM.pdf]

SUPPLEMENTARY METHODS

Micro-CT, histology, and histomorphometric analyses

For micro-CT, ankle and knee joints were dissected free of soft tissue, fixed overnight in 4% paraformaldehyde, and scanned at high resolution (10.5μm) on a VivaCT40 micro-CT scanner (Scanco Medical, Bassersdorf, Zurich, Switzerland) using 300 μs integration time, 55 kVp energy, and 145 μA intensity. 3D images were generated using a constant threshold of 275 for all samples. For histology and histomorphometric analyses, ankle and knee joints were fixed in 4% paraformaldehyde, decalcified in 14% EDTA. After dehydration, the joints were embedded in paraffin for paraffin sections or embedded in Tissue-Tek OCT compound for frozen sections for 3 levels (20 μm apart). The paraffin sections were stained with H&E for routine histology and for ALP activity to identify osteoblasts, TRAP activity to identify osteoclasts, Safrain O staining to identify cartilage. The frozen sections were mounted with Mounting Medium containing DAPI (Vector Labs, Burlingame, CA, USA) and images were captured with a Leica DM4000 fluorescence microscope.

Immunohistochemistry and immunofluorescence staining

The deparaffinized sections were subjected to heat mediated antigen retrieval, and blocked in H_2O_2 for 30 minutes, followed by PBS with 5% BSA and 0.2% Triton X-100 at room temperature for 30 minutes, and then stained overnight with primary antibody against F4/80 (Abcam, Cat#ab16911), RelA (Cell Signaling Technology, Cat#8242), NLRP3 (Abcam, Cat#ab214185), Caspase-1 (AdipoGen, Cat#AG-20B-0042), or IL-1β (R&D System, Cat#AF-401-NA) at 4℃. For immunohistochemistry staining, after rinsing with PBS for 15 minutes, tissues were

incubated with HRP-conjugated secondary antibody at room temperature. Sections were then washed and colors were developed with DAB (3,3′-diaminobenzidine). Next, hematoxylin was used as a counterstain. For immunofluorescence staining, tissues were incubated with goat anti-rat FITC (Proteintech, Cat#SA00003-11) or goat anti-rabbit Cy3 (Proteintech, Cat#SA00009-2) or donkey anti-goat Cy3 (Proteintech, Cat#SA00009-3) or goat anti-mouse Cy3 (Proteintech, Cat#SA00009-1) at room temperature. Slides were mounted with Mounting Medium containing DAPI (Vector Labs, Burlingame, CA, USA). All the images were captured with a Leica DM4000 fluorescence microscope.

Cell culture

RAW264.7 and HEK-293T cell lines were laboratory storage cells, and were tested for mycoplasma contamination before the experiment. RAW264.7 cells were maintained in DMEM supplemented with 10% fetal bovine serum (FBS), and 1% penicillin/streptomycin (P/S). HEK-293T cells were cultured in F12 medium, supplemented with 10% fetal bovine serum (FBS), and 1% penicillin/streptomycin (P/S). Bone marrow-derived macrophages (BMDMs) were isolated and generated from WT and NLRP3^{KO} mice as described previously [23]. Briefly, tibiae and femora were obtained and bone marrow (BM) cells were flushed out using α-MEM/2% FBS. After red blood cells were lysed, BM cells were cultured with M-CSF (R&D Systems, Minneapolis, MN) for 3 days in α-MEM/10% FBS to generate BMDMs. BMDMs was then primed with 20 ng/mL TNFα (R&D Systems, Minneapolis, MN) and cultured with or without 10μM Helenalin (MedChemExpress, USA) to block RelA activation.

Cell transfection

The recombinant lentiviral GV-309 vectors, encoding the miR-30a sequence was designed, synthesized and sequence-verified by Genechem Biotech Inc (Shanghai, China). MiR-30a and miR-433 mimics was purchased from Gene Pharma Co (China) and lipofectamine 2000 (Invitrogen) was used to transfect the miR-30a and miR-433 mimics. All of the transfection procedures followed the protocols of the manufacturer.

Quantitative real-time PCR

Total RNA was extracted from cell cultures, or from synovial tissues of ankle joints with TRIzol Reagent (Invitrogen). cDNAs were reversely transcribed with the PrimeScript RT Master Mix (Takara, Cat#RR036A) and subjected for RT-qPCR using specific primers. To test the miRNA expression, 500 ng total RNA from each sample was performed using a specific Reverse Transcription Kit from Ribobio (Guangdong, China). Relative expression of mRNA or miRNA was evaluated by the $2^{-\Delta\Delta Ct}$ method and normalized to the expression of β-actin or U6, respectively. The sequences of primer sets are shown in Supplementary Table 1 on line.

Western blot

Whole-cell lysates were lysed directly in RIPA buffer. Protein samples were quantitated by a BCA protein assay kit (Beyotime, Cat#P0012) and then separated by SDS-PAGE and transferred onto polyvinylidene diflouride (PVDF) membrane. Membranes were blocked in 5% milk in PBS for 3 hours at room temperature. After that, membranes were incubated at 4°C overnight with primary antibodies against NF-κB1 (Cell Signaling Technology, Cat#12540S), NF-κB2 (Cell Signaling Technology, Cat#4882S), RelA (Cell Signaling Technology, Cat#8242S), RelB (Cell

Signaling Technology, Cat#4922S), p-IκBα (Cell Signaling Technology, Cat#2859S), NLRP3 (Cell Signaling Technology, Cat#D4D8T), Caspase-1 (AdipoGen, Cat#AG-20B-0042), IL-1β (R&D System, Cat#AF-401-NA) and β-actin (Santa Cruz, Cat#sc-47778). The next day, after washing with PBST for 3 times, membranes were incubated with HRP-conjugated secondary antibody for 1 hour. Specific bands were developed using enhanced chemiluminescence (ECL) reagents (Tanon), visualized by Tanon-5200 Multi Chemiluminescent System (Tanon).

Luciferase reporter assay

MiR-30a and miR-433 binding sites on NLRP3 were searched and predicted by bioinformatics platforms. RAW264.7 and HEK-293T cells were seeded in 24-well plates respectively, and then transiently transfected with NLRP3 3′UTR-WT, NLRP3 3′UTR-Mut (Genechem, Shanghai, China), control or miR-30a mimics or miR-433 mimics using Lipofectamine 2000. After 24 hours, the cells were harvested and lysed in passive lysis buffer. Luciferase activity was measured using the Dual-Luciferase Reporter Assay System (Promega, Madison, WI, USA) according to the manufacturer's protocol. Renilla luciferase was used for normalization. The transfection experiments were performed in triplicate.

Intra-articular injection of AAV-miR-30a

MiR-30a or control sequences were cloned into the vector CV412-CMV-EGFP-MCS-WPRE, followed by infection to prepare adeno-associated virus (AAV). The preparation and purification of the AAV-miR-30a and AAV-miR-ctrl were performed by the Genechem Biotech Inc (Shanghai, China). As previously reported, arthritis develops in TNF^{TG} mice from 2 months old, and it

progresses with age [24]. Therefore, 2-month-old WT and TNF^{TG} male mice were used for the following intra-articular injections. After anaesthetizing the mice with isoflurane, 5μl of AAV-miR-30a solution (5×10^9 viral particles per μl) is injected into the joint cavity of the right ankle, whereas 5μl of AAV-miR-ctrl solution is injected into the left ankle, using a graded Hamilton syringe with 34-G needle. The injections were repeated monthly for 3 months. AAV transduction efficiency was determined by RT-qPCR as described above, and further confirmed by unstained frozen sections of ankle joint that were directly observed under fluorescence microscopy.

Statistical analysis

All in vitro experiments were performed at least 3 times and in vivo experiments were performed twice. During the period of analysis, identity information of each sample was sealed to avoid bias, and each value was double-checked by two examiners. All data are given as mean ± SD. Statistical analysis was performed using GraphPad Prism 8 software (GraphPad Software Inc, San Diego, CA, USA). Comparisons between 2 groups were analyzed using the 2-tailed unpaired Student's t-test. Comparisons among 3 or more groups were carried out using one-way ANOVA followed by Dunnett's post-hoc multiple comparisons. P values <0.05 were considered statistically significant.
