# Supplementary figures and images for: RelA/MicroRNA-30a/NLRP3 signal axis is involved in rheumatoid arthritis via regulating NLRP3 inflammasome in macrophages
Source: Cell Death Dis. 2021 Nov 8;12(11):1060. doi: 10.1038/s41419-021-04349-5 (PMC8575917; doi:10.1038/s41419-021-04349-5)

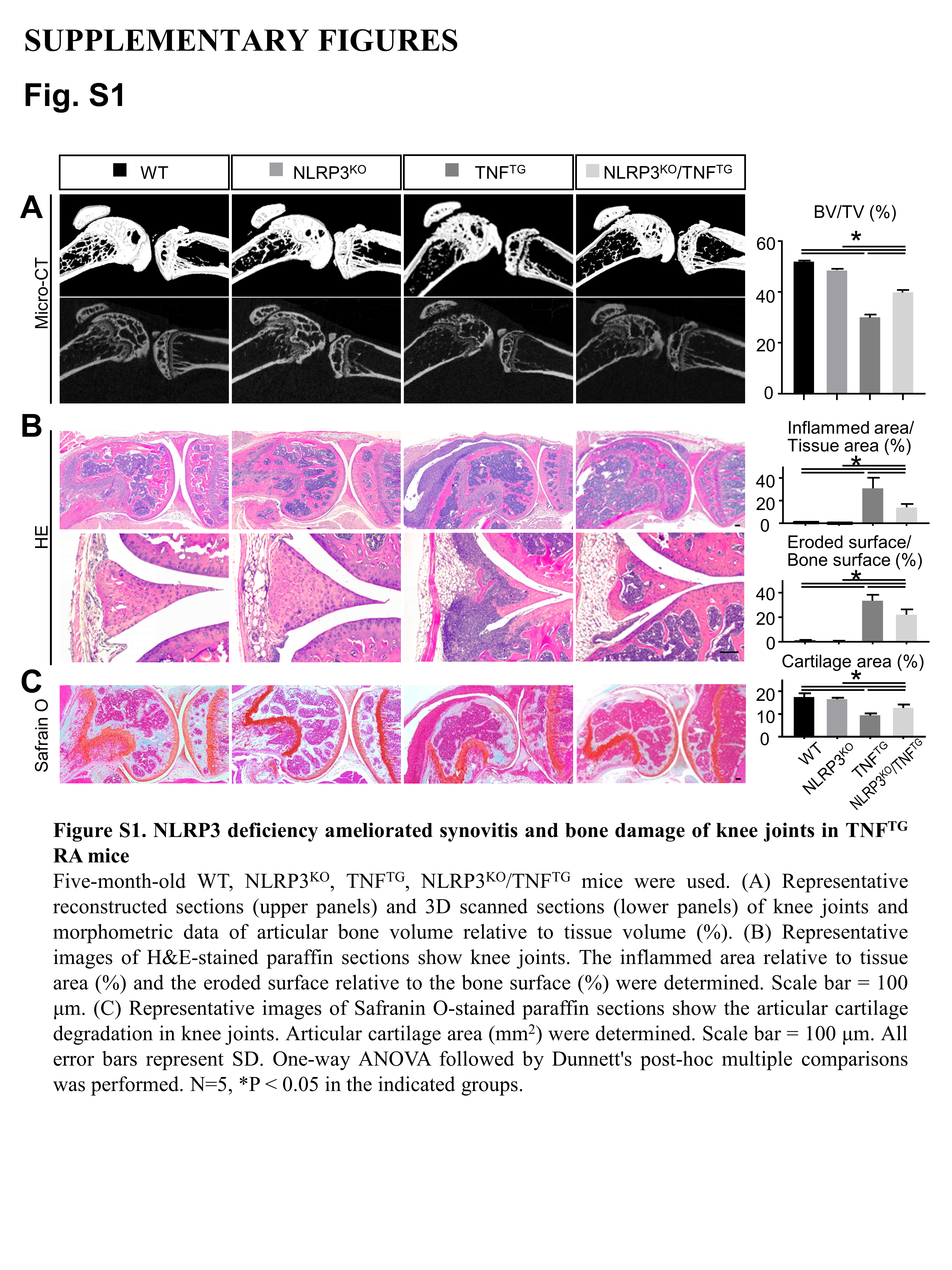

Supplement: Supplementary file 2 — Figure S1 [file 41419_2021_4349_MOESM2_ESM.tif]

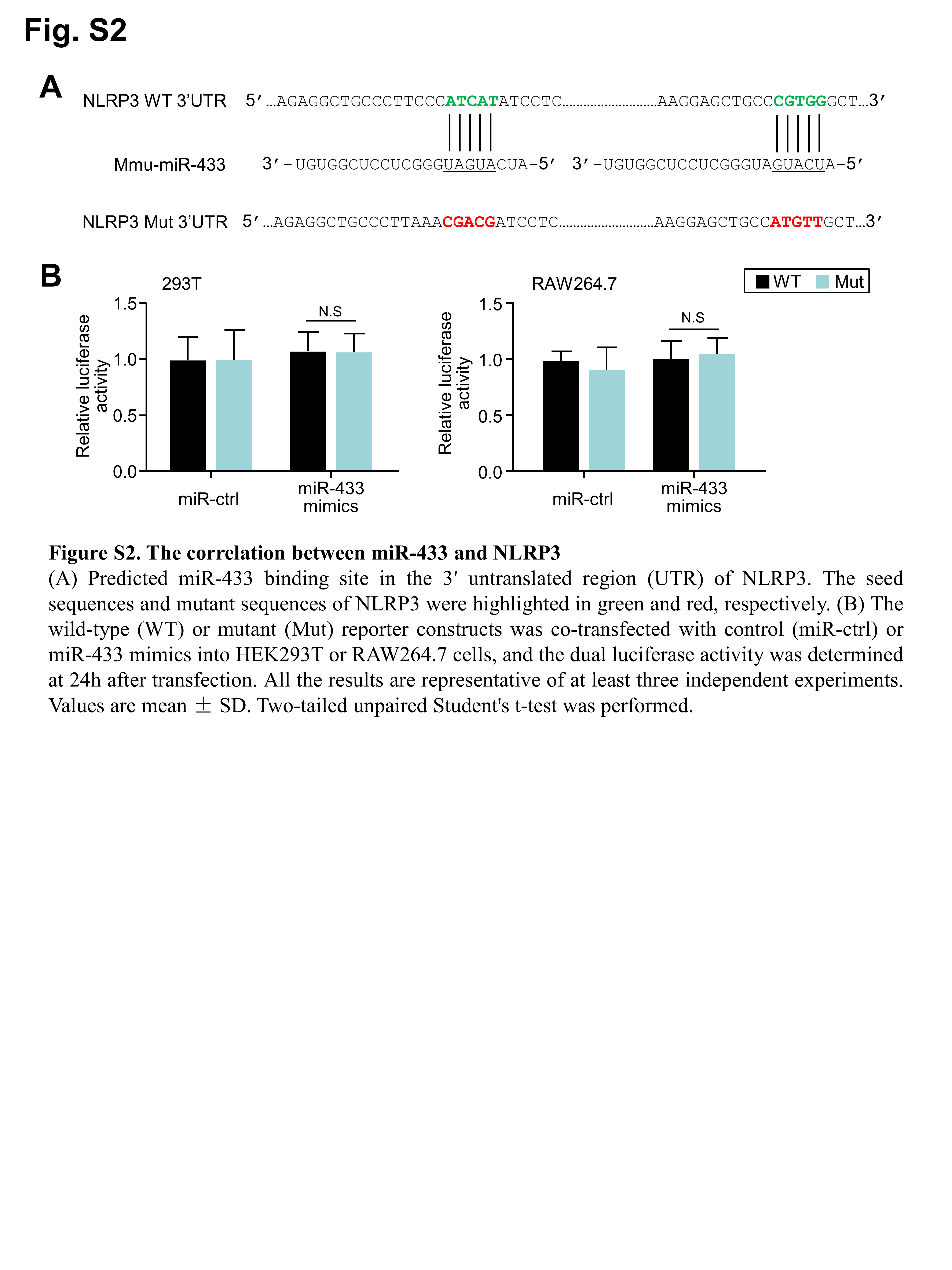

Supplement: Supplementary file 3 — Figure S2 [file 41419_2021_4349_MOESM3_ESM.tif]

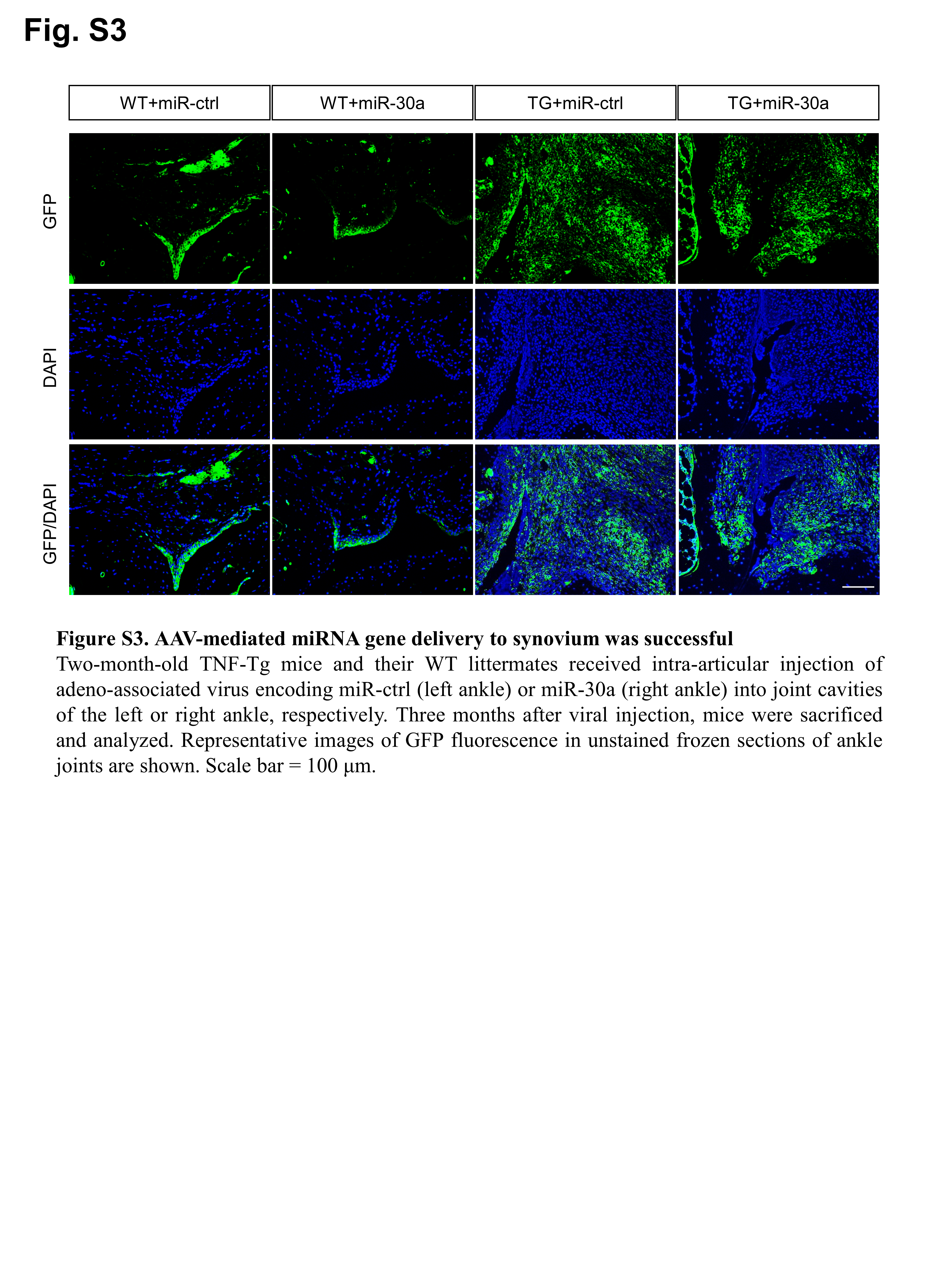

Supplement: Supplementary file 4 — Figure S3 [file 41419_2021_4349_MOESM4_ESM.tif]

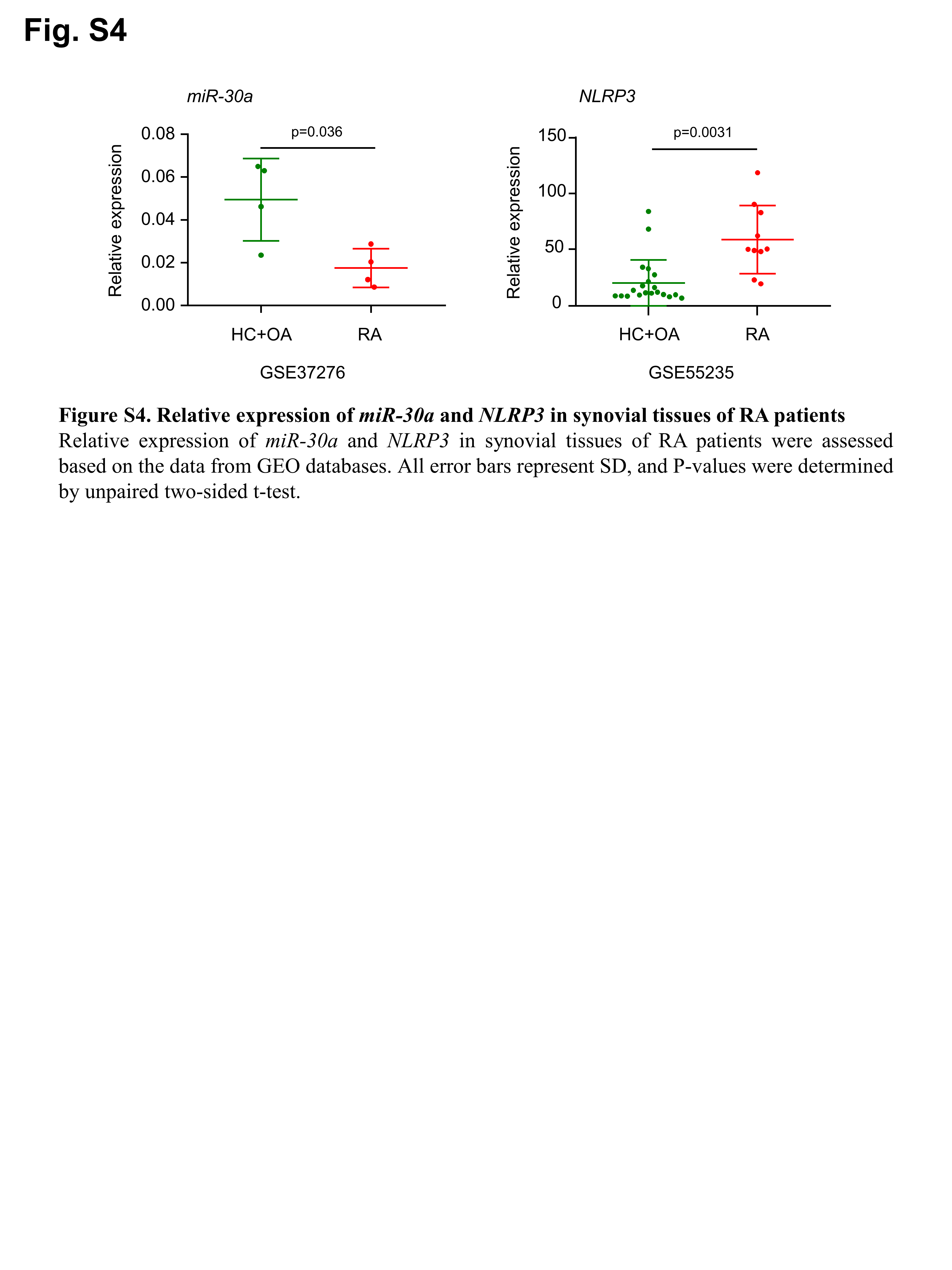

Supplement: Supplementary file 5 — Figure S4 [file 41419_2021_4349_MOESM5_ESM.tif]

Uncropped full Western blot scans of Fig. 4.

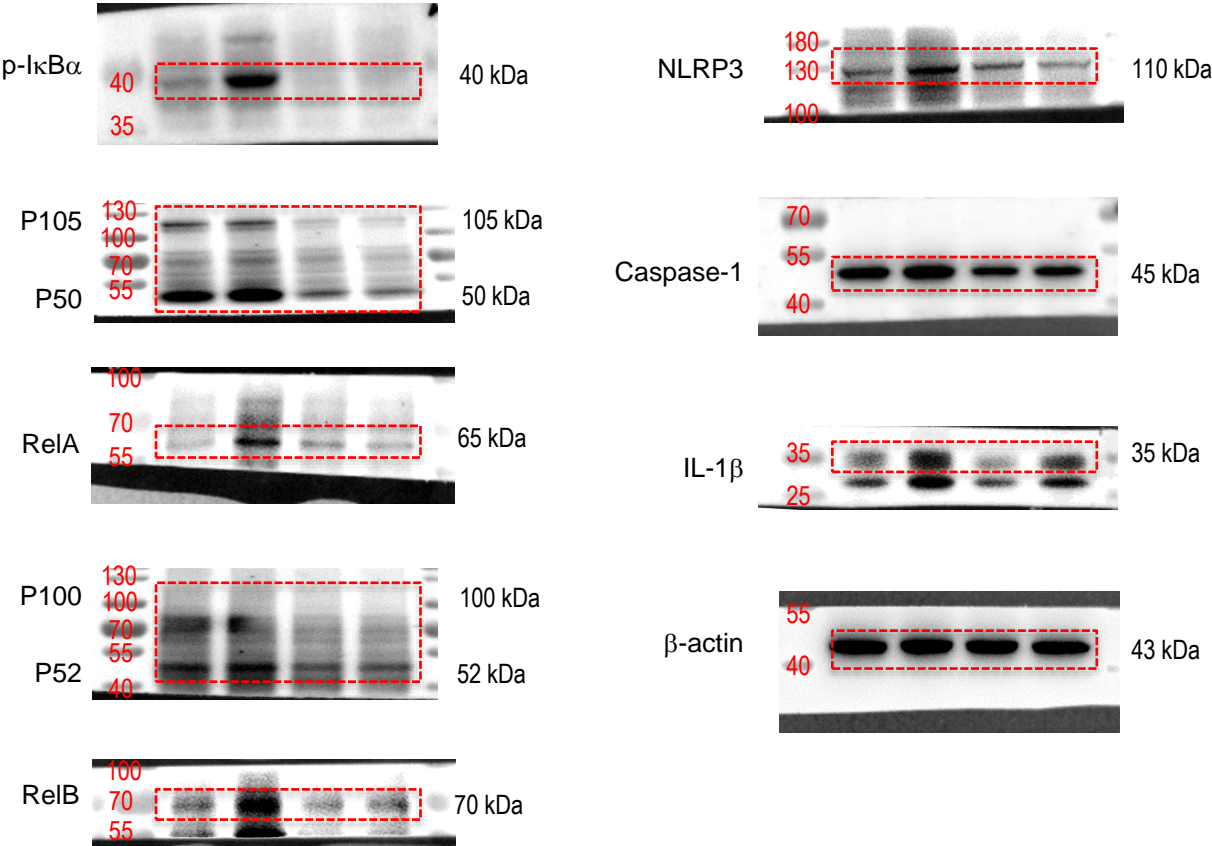

## Uncropped full Western blot scans of Fig. 5.

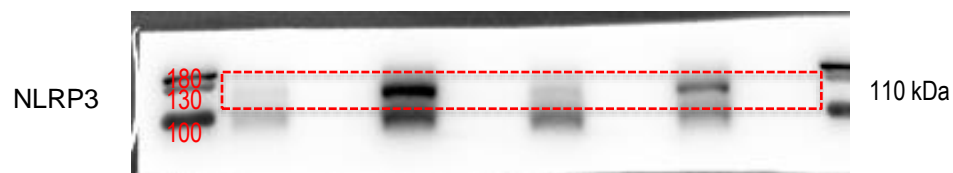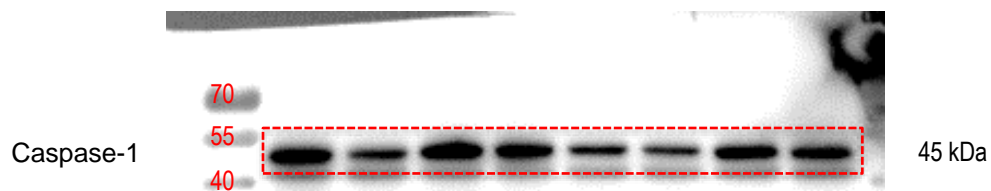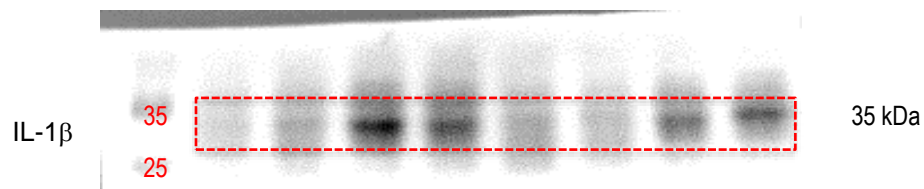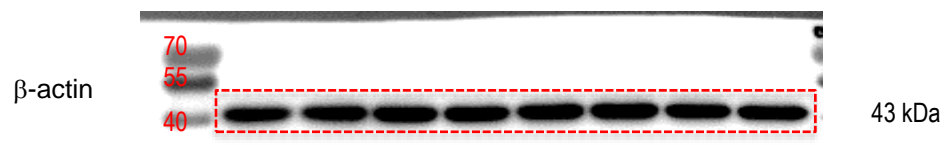

Supplement: Supplementary file 7 — Supplementary Information- Uncropped full Western blot scans [file 41419_2021_4349_MOESM7_ESM.pdf]
