# Supplementary material for: RelA/MicroRNA-30a/NLRP3 signal axis is involved in rheumatoid arthritis via regulating NLRP3 inflammasome in macrophages
Source: Cell Death Dis. 2021 Nov 8;12(11):1060. doi: 10.1038/s41419-021-04349-5 (PMC8575917; doi:10.1038/s41419-021-04349-5)
Supplement: Supplementary file 6 — Supplementary Table [file 41419_2021_4349_MOESM6_ESM.pdf]

SUPPLEMENTARY TABLE

Supplementary Table 1

Name	F/R	Sequences
Gapdh	F	5′-GGTCGGTGTGAACGGATTTG-3′
	R	5′-ATGAGCCCTTCCACAATG-3′
Nlrp3	F	5′-ATTACCCGCCCGAGAAAGG-3′
	R	5′-TCGCAGCAAAGATCCACACAG-3′
Il-1β	F	5′-GAAATGCCACCTTTTGACAGTG-3′
	R	5′-CTGGATGCTCTCATCAGGACA-3′
Caspase-1	F	5′-ACAAGGCACGGGACCTATG-3′
	R	5′-TCCCAGTCAGTCCTGGAAATG-3′
Nf-κb1	F	5′-ACAGCAGATGGCCCATACCT-3′
	R	5′-CATACATAACGGAAACGAAATCCTCT-3′
Nf-κb2	F	5′-CAGTGAGAAGGGCCGAAAGAC-3′
	R	5′-CAGGGGCAGGGAGAAGGAG-3′
Rela	F	5′-CTGCCGGGATGGCTTCTAT-3′
	R	5′-CCGCTTCTTCACACACTGGAT-3′
Relb	F	5′-GTGACCTCTCTTCCCTGTCACT-3′
	R	5′-TGTATTCGTCGATGATTTCCAA-3′

Table S1. The sequences of primers used.
